# Improving Delirium Assessments in Vanderbilt Pediatric and Pediatric Cardiovascular Intensive Care Units

**DOI:** 10.1097/pq9.0000000000000577

**Published:** 2022-07-13

**Authors:** H. Nur Eken, Kristina A. Betters, D. Catherine Fuchs, Heidi A. B. Smith, Stacey R. Williams

**Affiliations:** From the * Vanderbilt University School of Medicine, Vanderbilt University Medical Center, Nashville, Tenn.; † Department of Psychiatry, University of Pittsburgh Medical Center, Pittsburgh, PA.; ‡ Department of Pediatrics, Vanderbilt University Medical Center, Nashville, Tenn.; § Department of Psychiatry and Behavioral Sciences, Vanderbilt University Medical Center, Nashville, Tenn.; ¶ Department of Anesthesiology, Vanderbilt University Medical Center, Nashville, Tenn.; ‖ Pediatric Critical Care Unit, Monroe Carell Jr Children’s Hospital at Vanderbilt, Nashville, Tenn.

## Abstract

**Introduction:**

Delirium is a disturbance of attention and awareness that represents a change from baseline mental status. Accurate diagnosis of delirium is of paramount importance to improving the management of pediatric delirium in the intensive care unit. Despite ongoing education, inconsistencies in delirium assessments occur. Here, we aimed to determine the extent of the problem and increase compliance with delirium assessments.

**Methods:**

We collected preintervention data to assess baseline compliance of delirium assessments in the Pediatric Intensive Care Unit (PICU) and Pediatric Cardiac Intensive Care Unit (PCICU) at Monroe Carell Jr Children’s Hospital at Vanderbilt in November 2020. We executed 2 Plan-Do-Study-Act cycles with different interventions and collected data after each and approximately 1 year after the interventions. The first intervention consisted of virtual lectures on delirium assessments for the nursing staff. The second intervention included an educational handout and a new electronic medical record documentation tool.

**Results:**

Five hundred five individual nurse-patient encounters were assessed and collected throughout the project. The mean compliance of delirium documentation before the interventions was 52.5%. Target compliance after interventions was 70%. Mean compliance was 70% after cycle 1, 78% after cycle 2, and 86% in March 2022.

**Conclusions:**

Using pre- and postintervention data from chart reviews and nurse interviews regarding delirium screenings, we found that interventions targeting nurse education and EMR flowsheet improved compliance with delirium assessment and documentation in the PICU and PCICU. Future work should focus on assessing the clinical implications of this project in diagnosing and treating delirium.

## INTRODUCTION

Delirium is a disturbance of attention and awareness that represents a change from baseline mental status.^1^ It is a serious complication of critical illness and is associated with worse outcomes, increased length of stay, subsequent hospitalizations, and increased mortality rates.^2^

Recent research has described the risk factors, prevalence, and clinical correlates of pediatric delirium. Multiple studies have assessed the prevalence of pediatric delirium to be anywhere between 15% and 60%.^3^ Main risk factors associated with delirium include baseline developmental delay, need for mechanical ventilation, the severity of illness, coma, use of medications such as benzodiazepines and anticholinergics, and age younger than 5 years.^4^ Over 90% of the patients suffering from delirium have either the hypoactive or mixed subtypes that manifest with hypoactive behaviors instead of the more obvious agitated type of symptoms commonly reported as hyperactive delirium.^5^ Behaviors associated with hypoactive delirium can be more difficult to notice. Drowsiness, withdrawal, and inactivity characterize hypoactive delirium instead of the agitation and restlessness observed in a hyperactive delirium. Patients might make a little movement, not communicate or communicate slower and more quietly, and fail to respond to social interactions whereas awake.^6,7^ Behavioral disturbances, including combativeness and agitation associated with pediatric delirium, can harm patients and staff in intensive care units.^8^

Accurate diagnosis of delirium is of paramount importance to improving the management of pediatric delirium in the intensive care unit. Research groups have developed sensitive and specific bedside screening tools to monitor pediatric delirium.^8,9^ Smith et al. developed and validated the Pediatric Confusion Assessment Method for the ICU (pCAM-ICU) for developmental age 5 years or older and Preschool Confusion Assessment Method for the ICU (psCAM-ICU) for developmental age younger than 5 years. These tools screen for pediatric delirium objectively and interactively at the bedside. They are structured to standardize pediatric delirium assessments according to DSM criteria, focusing on core features of delirium such as inattention and sleep-wake cycle disturbances.^5,8^

The delirium team at Vanderbilt provided education to pediatric ICU faculty and staff preceding the initial implementation of the pCAM-ICU in 2011 and psCAM-ICU in 2016. They also provided training on the use of these screening tools to newly joining staff members and upon request to existing staff members. Despite these efforts, noncompliance has been noted in documentation and screening for delirium. This current quality improvement (QI) study aimed to increase compliance with pediatric delirium screenings in our Pediatric Intensive Care Unit (PICU) and Pediatric Cardiovascular Intensive Care Unit (PCICU) to 70% or above from the baseline measured in November 2020 through February 2021. In addition, it aimed to investigate the sustainability of interventions in 2022, a year from postintervention data collection.

## METHODS

### Setting

The study occurred in the medical and cardiac pediatric critical care units at Monroe Carell Jr. Children’s Hospital at Vanderbilt, a free-standing academic tertiary care children’s hospital with 339 beds (PICU: 42 beds, PCICU: 18−24 beds). Vanderbilt PICU serves children with primary medical conditions and those admitted after burn incidents or surgical procedures. In contrast, the PCICU serves children with congenital cardiac defects and arrhythmias and supports one of the highest volume heart transplant programs in the United States. Vanderbilt University Institutional Review Board determined that this QI project was not human subject research and did not require review and approval (IRB no. 202116).

### Interventions

**Fig. 1.**
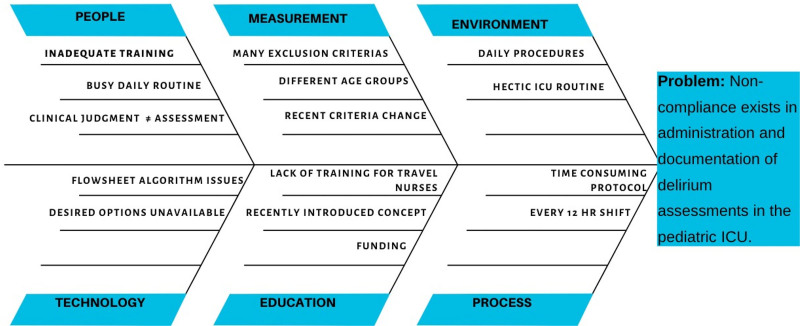
Fishbone diagram delineating root causes for delirium screening inaccuracies.

**Fig. 2.**
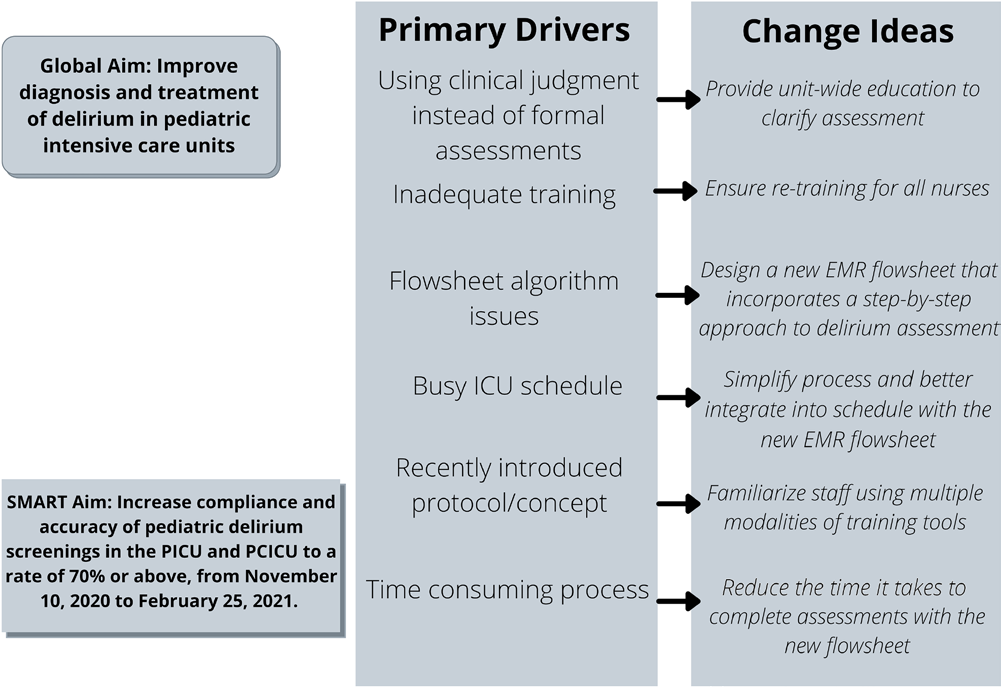
Key driver diagram of the pediatric delirium quality improvement initiative.

An interprofessional team of nurses, intensivists, psychiatrists, and anesthesiologists convened to assess the current delirium screening process and identify areas of improvement. The team created a key driver diagram and a fishbone diagram to outline contributors to the existing problem (Figs. 1 and 2). They then developed interventions informed by the key drivers of the problem as presented in the diagram. These interventions included: (1) training for all PICU and PCICU nurses consisting of virtual lectures on delirium epidemiology and pediatric delirium assessments, (2) educational handouts outlining the delirium screening process, and (3) a new, expanded nursing documentation flowsheet embedded in the electronic medical record (EMR). The team implemented and evaluated interventions using 2 consecutive Plan-Do-Study-Act (PDSA) cycles. The first cycle consisted of intervention 1, and the second cycle consisted of interventions 2 and 3. A team member (HNE) collected baseline data for compliance with delirium screenings between November 10, 2020, and November 22, 2020. She first reviewed the daily charting for all patients admitted to the PICU and PCICU for the presence of a pCAM-ICU or psCAM-ICU score. Then, the team interviewed the nursing staff to evaluate nurses’ compliance with bedside screenings to see if they followed the accurate pCAM-ICU and psCAM-ICU screening protocols. The team collected a total of 130 data points for baseline. They repeated this process after both PDSA implementation cycles to collect postintervention data, as well as approximately 1 year after the second intervention (March 2022) to demonstrate the sustainability of interventions.

It is important to note that during baseline data collection, guidelines designated pCAM-ICU and psCAM-ICU to screen patients 6 months and above. However, with the recent validation of the psCAM-ICU in infants <6 months of age,^10^ the team included data from patients including those <6 months of age during the postintervention phase.

On data collection dates, the team interviewed nurses who covered patients in the PICU (42 beds) or PCICU (24 beds) during a 12-hour shift, with a few exceptions. These included nurses who had to leave the service early for any reason and those who could not be interviewed due to time limitations at the end of the shift. Nurses caring for patients who transferred to a different unit or patients discharged shortly after admission and had incomplete documentation were also excluded. The team considered the screening to be complete when the documentation in the EMR stated “Delirium present,” “Delirium absent,” or “unable to assess,” and if the nurse noted compliance with the defined screening protocol for the same data point during the interview. Furthermore, the team confirmed using an age-appropriate tool based on patient age and developmental level for each patient screening, as noted in the EMR chart (neonate-up to 5 years: psCAM, 5–18 years: pCAM). If the nurse documented “unable to assess” for any patient screening, the team verbally confirmed the validity of the exclusion criteria. Valid exclusion criteria included a Richmond Agitation Sedation Scale (RASS) score equal to or <–4 or neurodevelopmental delay, including visual and hearing impairment, that prevented assessment. The team recorded this information in a REDCap (Research Electronic Data Capture^11,12^) form, where they included the date of assessment, patient age, whether the patient had a delirium assessment documented within the past 12 hours, and, if not, whether the patient met the criteria for exclusion, and presence of any barriers for assessment.

### Measures

The main outcome measure was the rate of correctly completed delirium assessments, calculated as the number of assessments accurately conducted (determined per the nurse interviews) and documented in the Epic EMR,^13^ divided by the total number of assessments. These interviews with the nurses were semistructured, where a team member asked standard questions about the nursing processes for screening the patients and documentation. In addition, each individual interview with a nurse was tailored based on the initial responses provided to better assess the completeness of the screening.

The process measure was the percentage of nurses engaged in the educational interventions of our team, although this work did not formally evaluate this measure. Balancing measures were twofold: The first was the potentially increased time required to complete the screenings due to the longer design of the new EMR flowsheet. The second was the qualitative feedback about interventions, measured by a survey sent to all PICU and PCICU nurses (see Supplemental Digital Content for the survey sent out to nurses regarding their opinions, http://links.lww.com/PQ9/A386).

### EMR Intervention

**Fig. 3.**
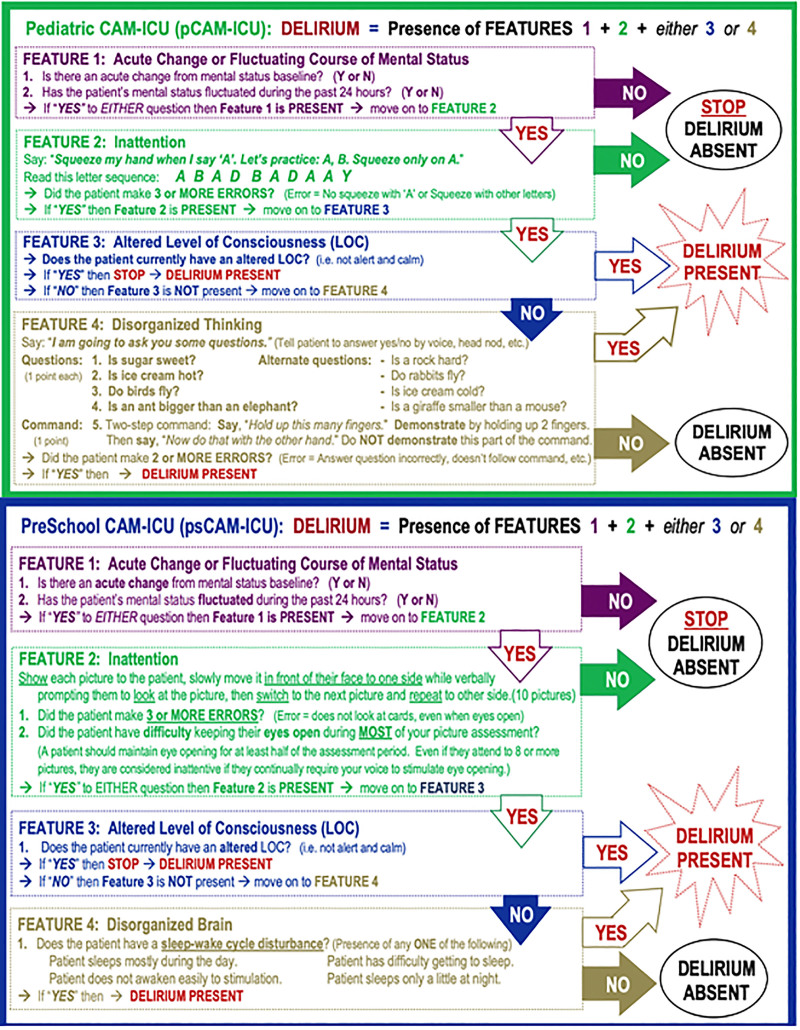
pCAM-ICU and psCAM-ICU pocket cards.

The study team worked with the hospital IT group to embed real-time guidance and education into the EMR to revise the current nursing delirium charting EMR flowsheets. Each step of the pCAM-ICU and psCAM-ICU assessments (Fig. 3) was built into the EMR with instructions on how to complete the assessment.^14^ This change eliminated the need to recall the full assessment from memory and ensured the completeness of the screening process in real time. Furthermore, advanced logic and decision support were built into the flowsheet to consider the screening algorithm. Such logic avoided unneeded assessments and automatically calculated the presence or absence of delirium based on nursing input. Once the revised EMR was complete, the research team worked with nursing educators to provide teaching sessions and materials to bedside nurses on the new flowsheet. Multiple stakeholders, including the delirium study team, the IT team, and nurse leaders from the pediatric critical care units, reviewed the revised EMR and made suggestions for improvement. The IT team then incorporated these suggestions to finalize the electronic documentation tool.

### Educational Interventions and Assessing Intervention Success

The project aimed to increase the percentage of delirium documentation from baseline compliance of 52.5% to 70% in 5 months. Baseline data collection took place between November 10, 2020, and November 22, 2020. The first intervention consisted of three 40-minute virtual lecture sessions (30-minute lecture time, 10-minute Q&A sessions) in December and January, excluding winter break (the last session on January 21, 2021). These virtual lectures were mandatory for all pediatric ICU nurses (100 nurses in the PCICU and 94 in the PICU). They provided an overview of delirium, focused on the importance of regular screenings, and described the planned EMR changes. Data collection for postintervention took place between January 29, 2021, and February 1, 2021.

Interventions in the second cycle consisted of an educational email handout for the nurses summarizing key points from the virtual lecture series, sent on the first week of February 2021. In addition, the IT team rolled out the new EMR flowsheet on February 4, 2021. The second round of audit and postintervention data collection took place between February 19, 2021, and March 9, 2021, and the final round of data collection assessing the sustainability of interventions was between March 2, 2022, and March 10, 2022. Two nurses enlisted as project champions, one from PICU and one from PCICU, reported to the delirium team what worked best and could improve the training and the EMR tool.

### Data Analysis

The team created a statistical process control chart to trend the compliance with delirium assessments per each data collection date to determine if the 2 interventions of the study affected observed outcomes. The team performed statistical testing and created the control chart using Microsoft Excel.

## RESULTS

The team collected data from 505 individual nurse-patient encounters throughout the project, with 130 data points obtained during the 5 days of the preintervention phase. In addition, they collected 79 data points after the first intervention, 109 after the second intervention, and 187 in March 2022, over a year after the interventions.

**Fig. 4.**
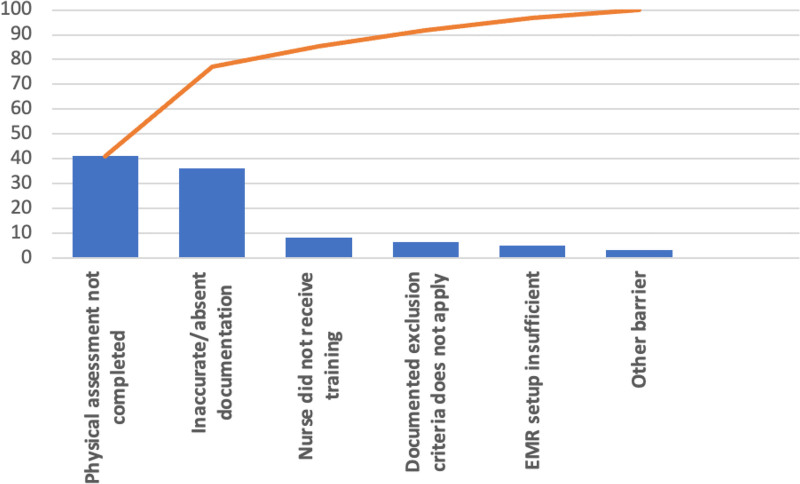
Pareto chart showing the percentage of errors from 6 categories in the preintervention phase (n = 61). The most common error type was no official assessment, followed by inaccurate/absent documentation, lack of training, wrong exclusion criteria, insufficient EMR setup, and other reasons.

**Fig. 5.**
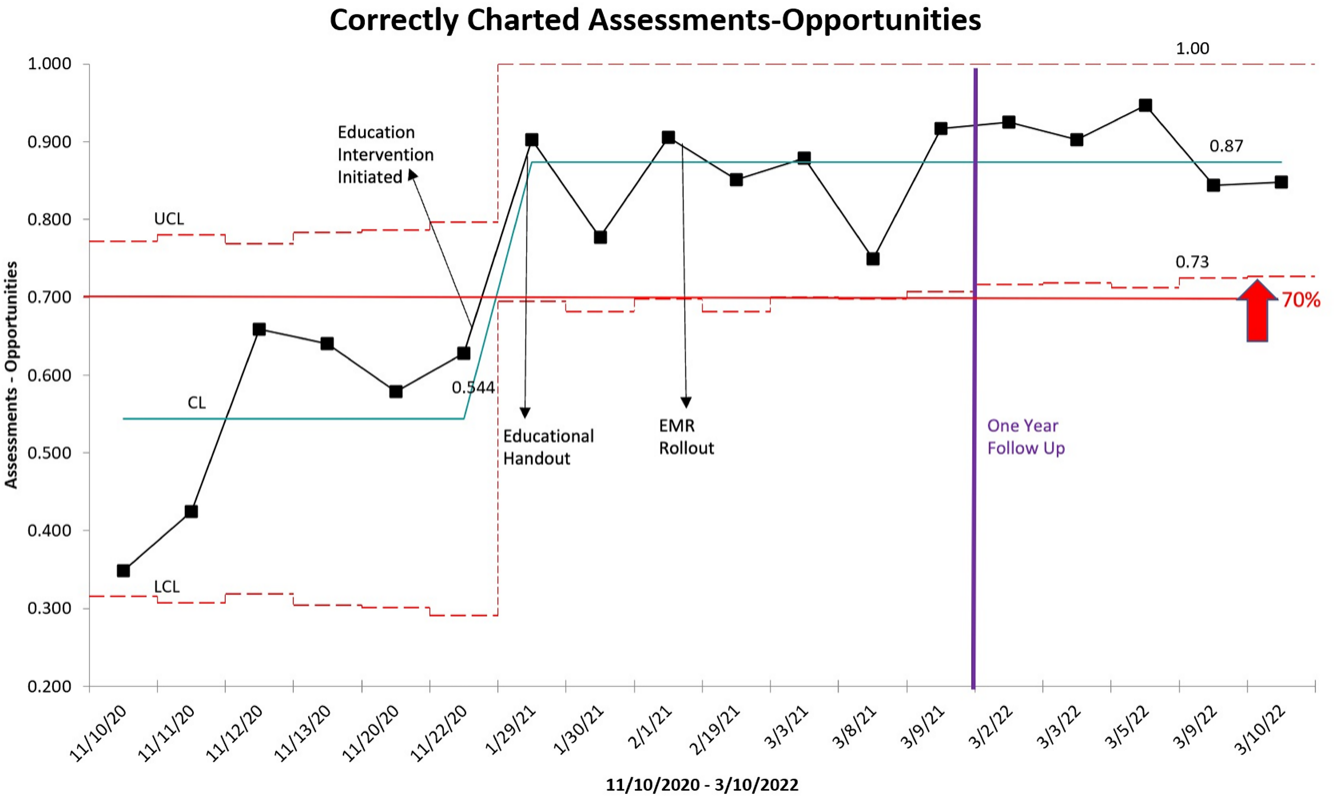
Statistical process control chart denoting the percentage of accurate delirium assessments pre- and postintervention. Final round of postintervention data were collected between March 2, 2022, and March 10, 2022. Each data point represents the percentage of completed assessments per day, divided by the total number of patients in the units qualifying for a delirium assessment that day. Mean compliance after the final intervention was 86%.

Mean delirium documentation and assessment compliance at the end of the preintervention cycle was 52.5%. Of the 130 preintervention data points, 61 represented inaccurate assessment or documentation. From most common to least common, error categories were as follows: No official assessment, which referred to nurses seeing the patient as generally alert and not conducting an official delirium screening (category a; n = 25; 40.98%); inaccurate or absent documentation, where the nurse documented the delirium assessment on the EMR but under the wrong assessment tool (eg, The nurse administered a pCAM but documented it under psCAM) or did not document at all (category b; n = 22, 36.07%). Other categories included lack of training, where the nurse reported not having received formal training (category c; n = 5, 8.20%); wrong exclusion criteria, where the nurse inaccurately skipped the assessment due to thinking that the patient met exclusion criteria (category d; n = 4, 6.56%). The final 2 categories were insufficient EMR setup, where the error happened because the nurse was unable to find the right documentation option on the EMR (category e; n = 3, 4.92%), and all other reasons (category f; n = 2, 3.28%) (Fig. 4). Mean compliance postcycle 1 was 70%, postcycle 2 was 78% (Fig. 5), and post-1 year was 86% (Fig. 5).

Finally, a survey was sent out to all PICU and PCICU nurses in March 2022 to better understand their perspectives on the interventions. Twenty percent of the actively working nurses (n = 39) completed the survey. The majority of the nurses from all experience levels reported that the delirium assessment was easier and quicker to use than the delirium cards, that the assessment took 1−5 minutes to complete, and that the new EMR assessment setup increased their knowledge regarding delirium (see Supplemental Digital Content for charts from the full survey http://links.lww.com/PQ9/A386).

## DISCUSSION

Using pre- and postintervention data from chart reviews and nurse interviews regarding delirium assessments, the QI team demonstrated that interventions targeting nurse education and EMR flowsheet upgrades significantly improved delirium screening and documentation compliance, exceeding 70%. Furthermore, compliance reached an even higher percentage in March 2022, with an average of 86% over a year after the PDSA cycles, demonstrating the sustainability of the interventions. The project’s success supports the importance of ongoing education and training for nursing staff on delirium assessment processes and the importance of a clear flowchart presentation on electronic medical records, which allows for easier, faster, and more accurate documentation. Although the initial cycle with an educational intervention showed the most dramatic improvement (52.5% to 70% versus 70% to 78%), this change also shows that training for nurses at the time of hire alone may not be sustainable. Therefore, continued education and user-friendly documentation tools in EMR are crucial to help sustain accurate delirium assessments.

During nursing interviews, the team noted several reasons for inaccuracies in documentation and screenings. The most common reason was omitting an official delirium screening when the nurse observed a patient to be alert and oriented, or thinking that completing a neurological assessment was sufficient to assess delirium. It was also common to have inaccurate documentation (eg, psCAM under pCAM or vice versa on EMR) or absent documentation. The team also noted that about 8% of the nurses reported never receiving training for delirium, with these nurses being predominantly “travel nurses” hired for a short period or float pool nurses who work in several different hospital units based on staffing needs. These findings demonstrate that although formal delirium education has been in place for all new ICU nurses, delirium education has not always been consistent for travel and float nurses. Keeping nurse training up to date throughout the year appears essential to ensuring accurate assessments and may underscore the need for deploying additional resources to train incoming new nursing staff. This finding was particularly notable in the last 2 years due to the increased percentage of travel and float nurses during the pandemic, highlighting the importance of continued and diligent training when healthcare systems experience significant stress.

In addition, improved compliance over 1 year after the interventions, with average compliance of 86% in March 2022, demonstrated the efficacy of improvements to EMR setup. The original EMR flowsheet had several issues that made accurate and effective documentation of delirium screenings difficult. First, the screening logic for delirium embedded in the pCAM and psCAM screenings lacked clear and automated flow logic (an example would be a flow logic where answer 1 is a “Yes,” move on to answer 2). Second, the setup was not user-friendly, and there was no clear space for documenting cases that met exclusion criteria or reasons for exclusion. The new EMR setup improved upon these issues, making the flow logic clear that even without pCAM and psCAM assessment cards, nurses could complete assessments in real-time as they were documenting. Most nurses in the PICU and PCICU with varying experience levels reported that the new EMR was easier and quicker to use than the alternative assessment methods (reference Supplemental Digital Content for nursing survey results http://links.lww.com/PQ9/A386). Even 1 year after the interventions, the continued improvement in compliance further highlights the importance of a user-friendly, clear EMR flowsheet that embeds the delirium assessment algorithm, allowing the nurses to complete their assessment and documentation in tandem.

Previous research has focused on introducing clear and automated tools in electronic medical records to improve delirium diagnosis and treatment.^15^ It also highlighted the role of other interventions involving bioinformatics technology, including automated risk stratification tools, to predict delirium in patients. The significant role of EMR-based interventions in quality improvement in hospital settings in general, and ICU in particular, has been widely demonstrated.^16–18^ This quality improvement project contributes to the existing literature by showing that one EMR-related intervention, introducing protocol flowsheets, improves compliance with delirium assessments. Future studies might focus on additional interventions that introduce newer automated systems into EMRs to aid healthcare professionals in conducting and documenting easily and efficiently.

Prior quality improvement projects focusing on delirium screenings showed improvement in delirium detection and treatment with an ultimate decrease in delirium prevalence.^19^ Our study thus extends the previous literature demonstrating the efficacy of educational programs and ICU bundles in increasing awareness of and preparation to assess and treat delirium.^20^ We hope that the significant improvement in delirium assessment compliance we observed in this QI study will lead to better detection and management of pediatric delirium. Future work should assess the clinical implications of this project—specifically, how increased accuracy in delirium screening may lead to more timely diagnosis and treatment for patients with delirium. Prior validation studies for pCAM and psCAM at Vanderbilt identified the delirium rate of infants at the hospital to be 44−47% and 13.2% for the 2 studies, respectively.^5,21^ We hope that clinicians and health systems can now establish the rate of delirium outside of delirium tool validation studies. In addition, knowledge of morbidity and mortality associated with delirium can significantly impact patient outcomes.

Limitations include the subjective nature of interviews with the nursing staff. For instance, it is possible that some nurses who endorsed completing the assessment accurately might not have done the assessment differently from the nurses who reported using only clinical judgment and completing the full assessment. However, we tried to minimize this potential bias by conducting thorough interviews, understanding nurses’ assessment and exam styles, interviewing the same nurses on different days, and consulting other team members for reliability. Furthermore, we collected our baseline data within 12 days due to time constraints per the external deadlines set by the IT department. However, in this relatively short period, we collected over 100 data points from thorough interviews with nurses and chart reviews. We built on this baseline with 3 distinct periods of postintervention data collection. In addition, the percentage of all the patients we collected data on was lower during the baseline than after the interventions. This fact is largely because psCAM used to be only validated for ages 6 months and older. These exclusion criteria changed before our post- intervention data collection. Our study was also limited to 1 institution. However, the QI methodology used is standardized and generalizable to other institutions. Prior QI studies employing educational interventions have shown effectiveness in different institutions,^19,22,23^ suggesting that other institutions can employ our interventions to achieve similar results.

Despite the growing body of literature highlighting the prevalence and impact of pediatric delirium, knowledge gaps and inaccuracies in assessments remain.^24^ Our study contributes to research focusing on improving delirium assessments and addressing preexisting gaps in understanding pediatric delirium, with the ultimate goal of earlier identification and treatment of delirium in pediatric critical care settings and beyond.

## CONCLUSIONS

In summary, we demonstrated that interventions focusing on nurse education and optimizing EMR data entry improved screening and documentation of delirium in the pediatric ICU setting. Continued education and training on screening can significantly improve delirium assessment processes, especially given the typical turnover of nursing staff and the addition of new staff. Future work should explore whether such interventions lead to faster and more accurate diagnoses and improve management of delirium and outcomes in pediatric ICU patients.

## DISCLOSURE

The authors have no financial interest to declare in relation to the content of this article.

## ACKNOWLEDGMENTS

We want to thank the pediatric ICU nurses for their continued help and support during our data collection and implementation.

## Supplementary Material


