# Supplementary material for: Improving Delirium Assessments in Vanderbilt Pediatric and Pediatric Cardiovascular Intensive Care Units
Source: Pediatr Qual Saf. 2022 Jul 13;7(4):e577. doi: 10.1097/pq9.0000000000000577 (PMC9278948; doi:10.1097/pq9.0000000000000577)
Supplement: Supplementary file 1 [file pqs-7-e577-s001.pdf]

Part A: Charts from nursing survey exploring their perspective on the EMR changes, including the changes in nursing time burden. A total of 39 nurses (20% of all active nurses at the time) filled out the main survey.

What unit and shift do you work? n=39

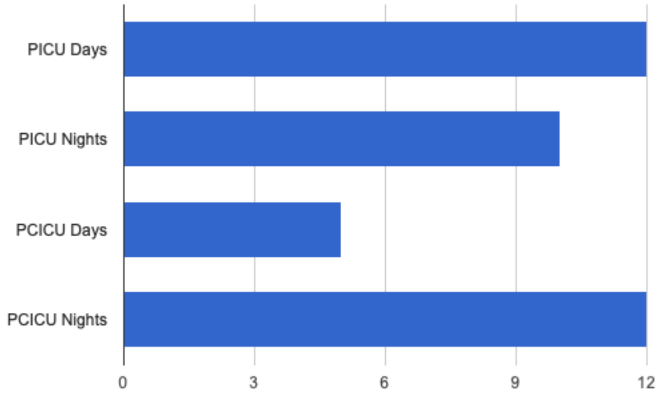

Years of experience n=39

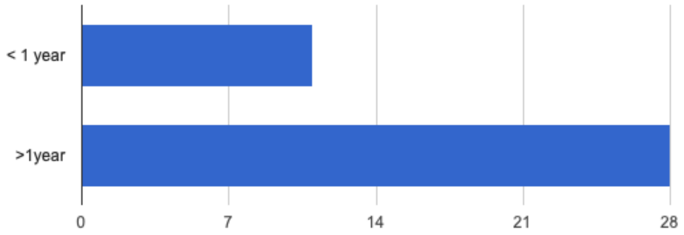

n=11 for next several for nurses who started <1yr

For nurses who work <1yr what month did they start?

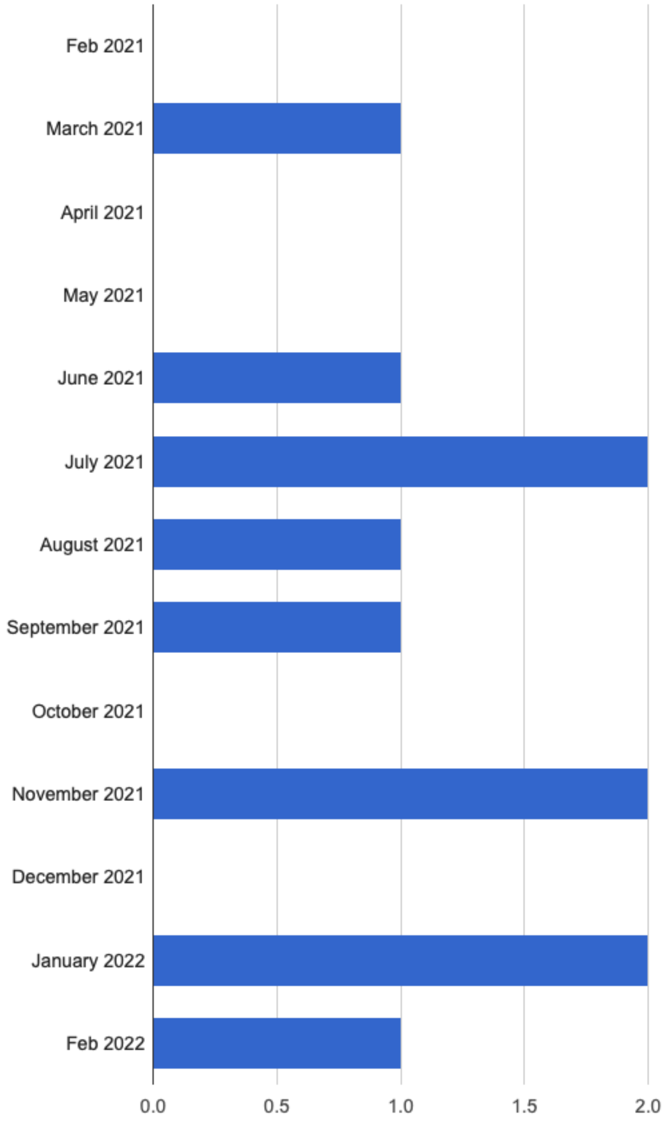

Did you receive any formal training on delirium?

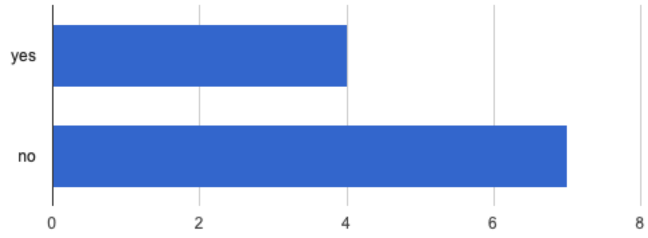

Was the EMR delirium assessment easy to use?

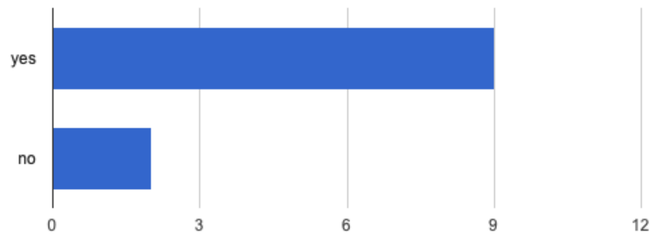

Do you think the EMR delirium assessment has increased your knowledge regarding delirium?

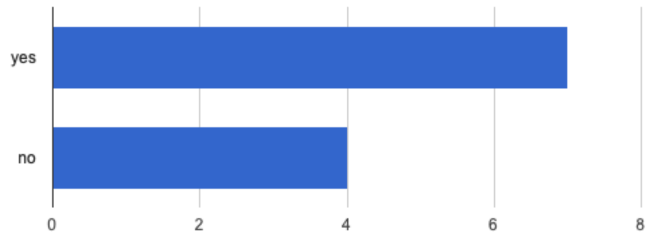

Do you think the EMR delirium assessment has helped make you more confident in performing delirium assessments?

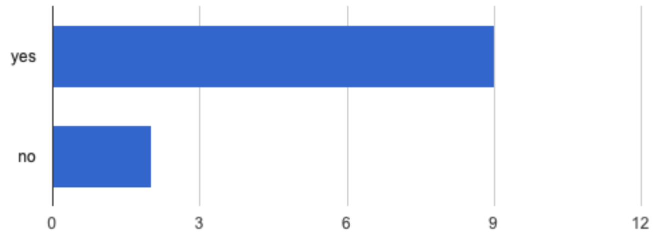

n=28 for the next set of questions for nurses >1yr of experience

Do you find the EMR delirium assessment easy to use?

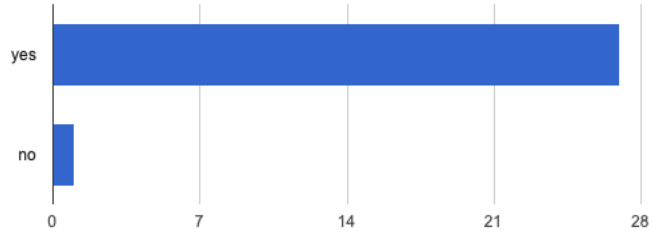

Do you find the EMR delirium assessment easier to use than the delirium cards?

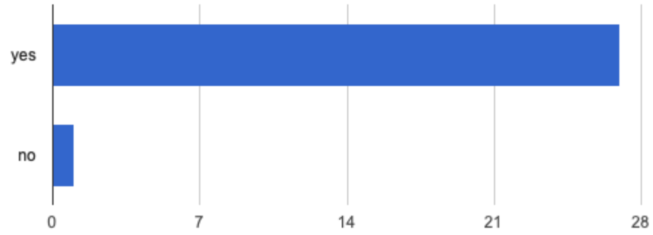

Do you find the EMR assessment to be quicker than the delirium cards?

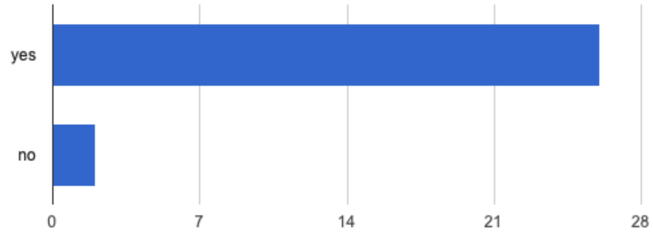

How long does the EMR delirium assessment take to complete?

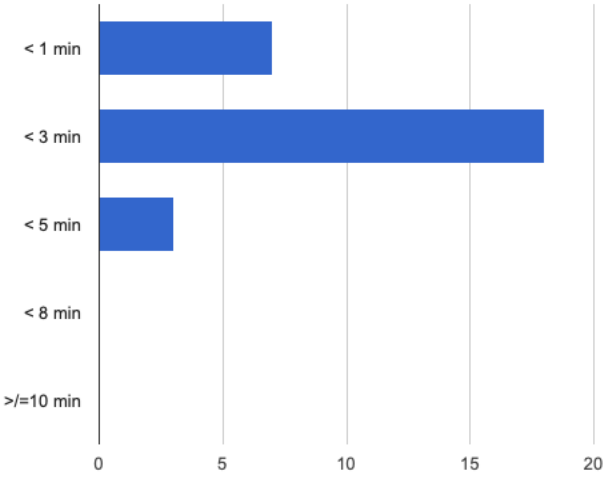

Do you think the EMR delirium assessment has increased your knowledge regarding delirium?

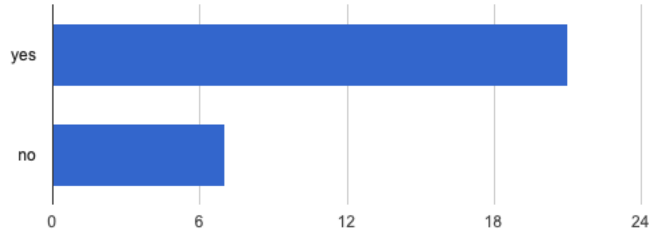

Do you think the EMR delirium assessment has helped make you more confident in performing delirium assessments?

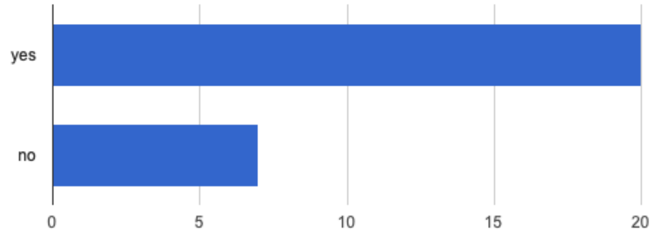

Part B. Linear control charts with full list of dates. First chart includes data from 2020 and 2021, second chart also incorporates data from final round of data collection in 2022.

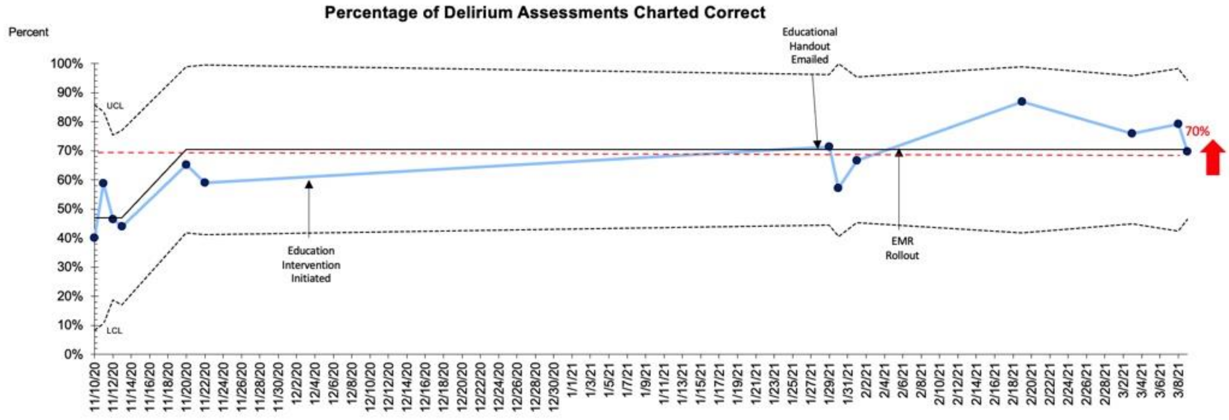

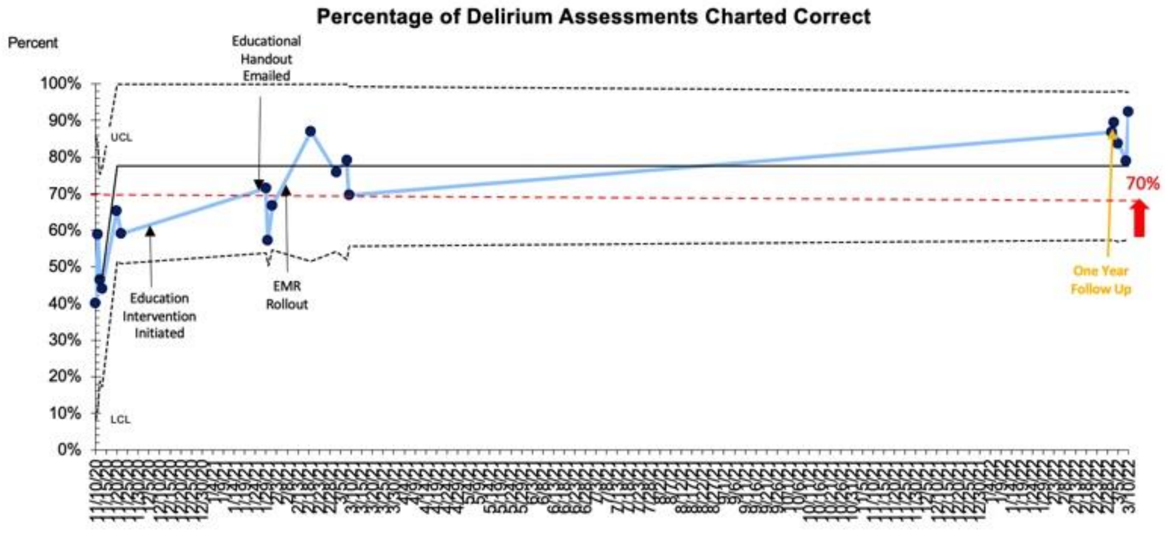
Percentage of Delirium Assessments Charted Correct
Percent
Educational
Handout
Emailed
UCL
LCL
Education
Intervention
Initiated
EMR
Rollout
70%
One Year
Follow Up
